# A Dynamic Hanging-Drop System for Mesenchymal Stem Cell Culture

**DOI:** 10.3390/ijms21124298

**Published:** 2020-06-16

**Authors:** Shu-Wei Huang, Shian-Chiuan Tzeng, Jem-Kun Chen, Jui-Sheng Sun, Feng-Huei Lin

**Affiliations:** 1 Department of Biomedical Engineering, National Taiwan University, Taipei 10617, Taiwan; judyya1022@gmail.com (S.-W.H.); papayohi@gmail.com (S.-C.T.); 2 Department of Materials Science and Engineering, National Taiwan University of Science and Technology, Taipei 10607, Taiwan; jkchen@mail.ntust.edu.tw; 3 Department of Orthopedic Surgery, National Taiwan University Hospital, Taipei 10002, Taiwan; drjssun@gmail.com; 4 Director, Institute of Biomedical Engineering and Nanomedicine, National Health Research Institutes, Miaoli 35053, Taiwan

**Keywords:** 3D culture, spheroid, hanging drop, microfluid

## Abstract

There have been many microfluid technologies combined with hanging-drop for cell culture gotten developed in the past decade. A common problem within these devices is that the cell suspension introduced at the central inlet could cause a number of cells in each microwell to not regularize. Also, the instability of droplets during the spheroid formation remains an unsolved ordeal. In this study, we designed a microfluidic-based hanging-drop culture system with the design of taper-tube that can increase the stability of droplets while enhancing the rate of liquid exchange. A ring is surrounding the taper-tube. The ring can hold the cells to enable us to seed an adequate amount of cells before perfusion. Moreover, during the period of cell culture, the mechanical force around the cell is relatively low to prevent stem cells from differentiate and maintain the phenotype. As a result of our hanging system design, cells are designed to accumulate at the bottom of the droplet. This method enhances convenience for observation activities and analysis of experiments. Thus, this microfluid chip can be used as an in vitro platform representing in vivo physiological conditions, and can be useful in regenerative therapy.

## 1. Introduction

Cell therapy is a technique which is used to reconstruct the human body structure for treating various diseases [1]. According to the cell types used, cell therapy can be divided into immunotherapy and stem cell transplantation [2]. In cell therapy, immune cells are collected from a patient, including the natural killer cells, dendritic cells, and T cells. The National Institute of Health (NIH) has listed immune cell therapy as a model for cancer treatment. A stem cell transplant is based on the process of introducing stem cells into a tissue to treat a disease that destroys stem cell or the bone marrow. Stem cells are cells which have the ability to renew themselves and can differentiate into various cell types—such as blood cells, and cells of the brain, bones, and organs of the human body under certain physiological or experimental conditions—and are widely used in tissue engineering research [3]. After cell passaging and proliferation, these cells are injected back into the patient [4]. However, in both stem cell transplantation and immunotherapy, large numbers of cells are required for preparing a matrix that can be injected back into a patient’s body to repair damaged tissue [5].

Traditionally, two-dimensional (2D) cell culture is a common method for proliferate cells. Most of the previously reported cell-based studies have been carried out under 2D conditions. However, most of the cells in an organism exist in a three-dimensional (3D) microenvironment [6]. The 2D culture method does not fully reflect the actual physiology of tissues, whereas the 3D culture mimics the specificity of the microenvironment during cell growth [7]. Therefore, studies of 3D cell culture have gained attention. To mimic the physiological conditions during cell development, cells should be allowed to aggregate into a spheroid form without external influences [8].

Cell spheroids are formed by self-assembly. In the past decade, many spheroid fabrication methods have been reported, such as hanging-drop methods [9], use of microfluidic devices as auxiliary tools [10], and culturing cells on low-adhesive substrates [11]. The hanging drop technique is a well-established method used for cell culture and has been used to create a 3D microenvironmental niche for developing tissues. In the traditional hanging-drop method, cells in the culture medium suspension are placed on the underside of petri dish lids. The cells accumulate at the tip of the drop, spontaneously aggregate, and form spheroids [9,12]. However, this method is limited by the difficulty in exchanging the culture medium, thereby preventing cells from being cultured for more than 3 days. Furthermore, cells within the spheroids are subject to diffusive oxygen and nutrition supply. Additionally, it is critical to prevent the necrotic damage to the cells at the core of the spheroid and to control the size and composition of spheroids [13]. Thus, an efficacious method that does not cause cell damage must be compatible with scaling-up process.

Microfluidic devices have very broad applications in biological assays from simple chemotaxis assays and it allowed to control the physiological relevance of the 3D culture by perfusion flow through micrometer size channels [14]. The culture systems can mimic cell-specific physiological conditions, like shear stressor chemical gradient [15,16,17]. Reports of stem cells cultured on the microfluid systems have been focused on the culture methods, cell differentiation, drug screening, and therapeutics [18,19,20]. Despite the development of many microfluid technologies combined with hanging-drop for cell culture [10,21,22,23], the common problem with these devices is that the cell suspension is centralized introduced at the inlet. The disadvantage of this approach is the number of cells in each microwell not regularize, which would cause the diameter of the spheroid is inconsistent. On the other hand, most existing devices could not interrogate the spheroids on the same device. Those devices often require an amount of manual sample handling, which can be prone to variability and error.

In this study, we developed a microfluidic-based hanging-drop culture system which enables cells to aggregate into spheroids spontaneously. Another feature of the design in this study, we can seed the number of cells we need in each microwell, which can load cells to avoid cells falling or being washed away by the flow. In order to ensure cell viability, a culture system that mimics the microenvironment of cell growth is needed. In the meantime, the culture medium is allowed to be changed to maintain the nutrient levels needed for cell proliferation.

## 2. Results

To verify the effectiveness of our microfluid chip in cell culture, we used Wharton’s jelly mesenchymal stromal cells (WJ-MSCs) as a model. Most cells can be cultured in a 2D environment but may show a limited proliferation rate. Another method for culturing cells is seeding the cells into a 3D space built from a synthetic scaffold, which may result in a loss of the cell phenotype. We used a microfluid chip with a peristaltic pump to provide continuous medium replenishment. This 3D culture system can prevent loss of the cellular phenotype and provide an active environment for cell spheroid growth which can be used in cell therapy.

### 2.1. Microfluid Chip Concept

**Figure 1:**
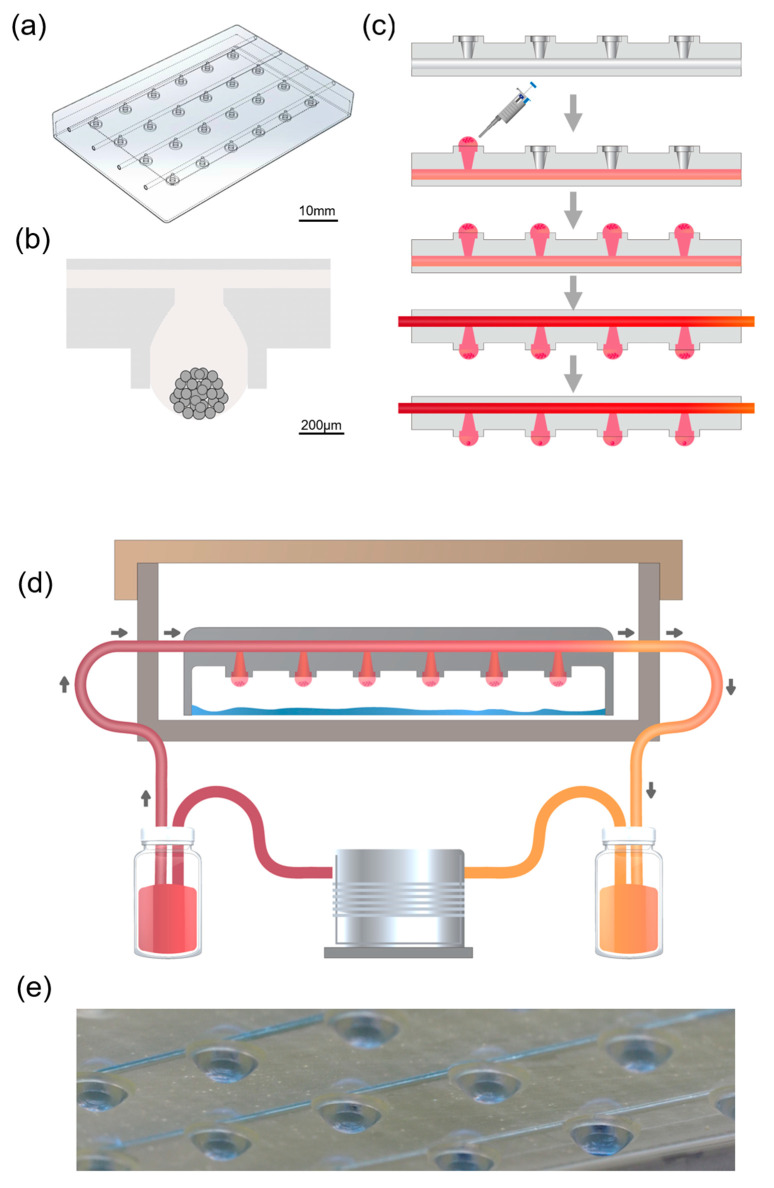
Schematic illustrations of the general concept and strategy for the microfluidic-based hanging-drop culture system. (**a**) Microfluidic-based hanging-drop culture chip. (**b**) Cell scattered at the bottom of the drop after seeding and aggregated into irregular shape spheroids cell in taper-tube. (**c**) The workflow of cell culture chip: fill the medium into the channel of the chip, add the cell into the tray, turn over the chip, then connect the chip with the culture system to start the culture of the spheroid. (**d**) Incubate the cells in the microfluid-based hanging-drop culture system for proliferation. (**e**) Photograph of the chip filled with water showing equal-size drops.

The microfluidic hanging drop chip is composed of 4 × 6 opening wells on a poly-methylmethacrylate (PMMA) slide which has been shown in Figure 1a. This chip was loaded into a CO_2_ laser scriber to ablate the patterns onto PMMA sheets. The microfluid chip consisted of the cell culture device and a unit that provided the fluid. The top layer of the chip contained four sets of medium channels with 1 mm diameter which connect inlets, and outlets. When liquid was applied through an inlet, it was drawn through the network by capillary forces along the rims. The bottom of the chip contained six hanging culture areas connected by one medium channel though a taper-tube, the initial structure of tubes with a 5 mm diameter and protruded to various conical degrees. Drops that were formed below the taper-tube areas increased with the liquid pressure. The drop size was determined by the surface tension at the liquid–air interface and lateral distance of the rims at the bottom. The liquid–air interface prevented cell adhesion in the microfluidic channels. This system allowed for gas exchange or nutrition exchange during incubation. The schematic diagram of the microfluidic-based hanging-drop culture system is as Figure 1d,e.

### 2.2. Drop Formation and Solution Exchange

**Figure 2:**
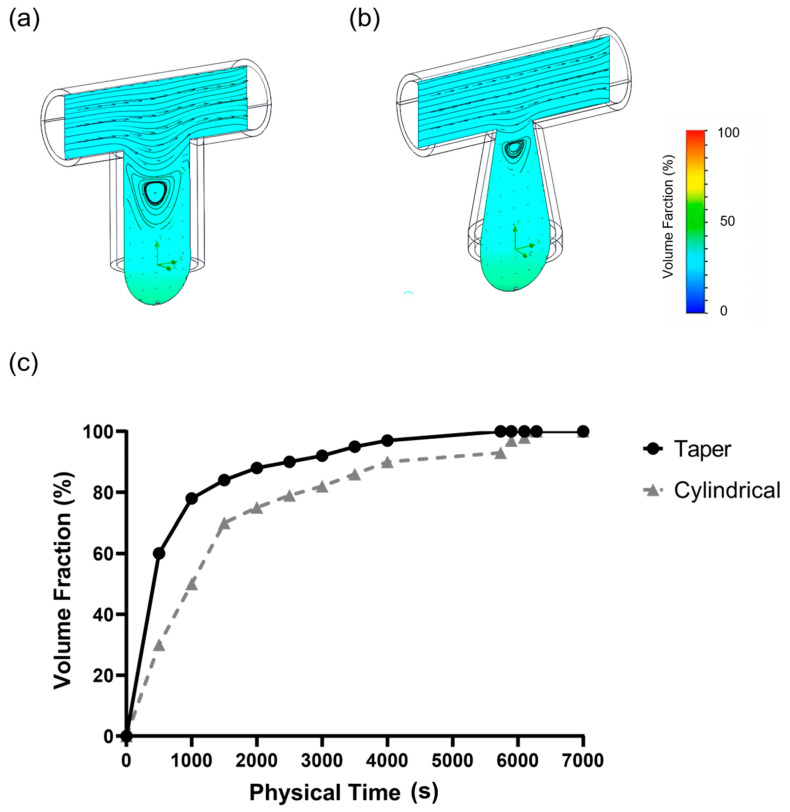
Model of computer simulation microfluid chip medium exchange: (**a**) Cylindrical tube microfluid chip, (**b**) taper-shaped microfluid chip, and (**c**) result of the medium exchange rate through computer simulation.

In this study, the 3D modeling method was applied to simulate the medium exchange process of a single culture tray with an inlet flow rate of 0.5 cc/h and two-phase flow simulation in a finite volume method. The effectiveness and the efficiency of the medium exchange process were determined by observing the volume fraction changes in moisture in the simulation area at different time points. The medium reached the bottom of both types of culture trays by natural convection and free diffusion under the condition of 0.5 cc/h medium exchange. As shown in the curve of the water volume fraction as a function of time, the taper-shaped tray was relatively efficient. At an influent time of 6284 s, the volume fraction of the new medium exchange at the bottom of the cylindrical tray reached 99%, whereas in the taper-shaped tray, this progress occurred at 5375 s (Figure 2).

**Figure 3:**
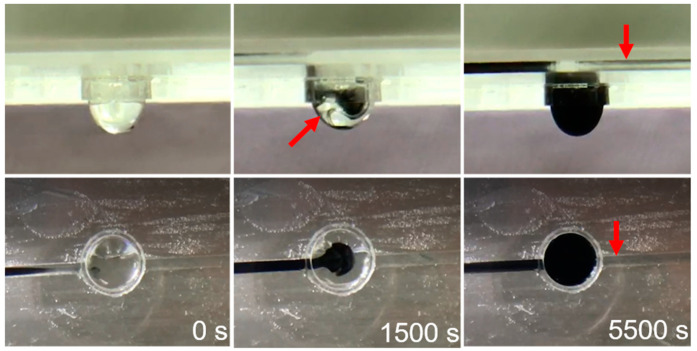
Schematic diagram of medium exchange, side view, and top view: at 0 s, the droplet is colorless. To 1500 s, new medium (black) infusion, we can see the liquid agitated and merged gradually. Until 5500 s, the droplet color changed into black and some medium toward the outlet.

We also observed the movement of methyl blue to verify medium exchange. As shown in Figure 3, the microfluid chip was initially filled with water, and then water containing methyl blue was injected into the chip from the inlet at a rate of 0.5 cc/h. Water stained with methyl blue filled the tube form inlet by the capillary phenomenon then replaced the original water in the tray because of its different density and through free diffusion. At the same time, the peristaltic pump discharged the liquid from the outlet at a constant rate to achieve liquid exchange.

### 2.3. Droplet Stability

**Figure 4:**
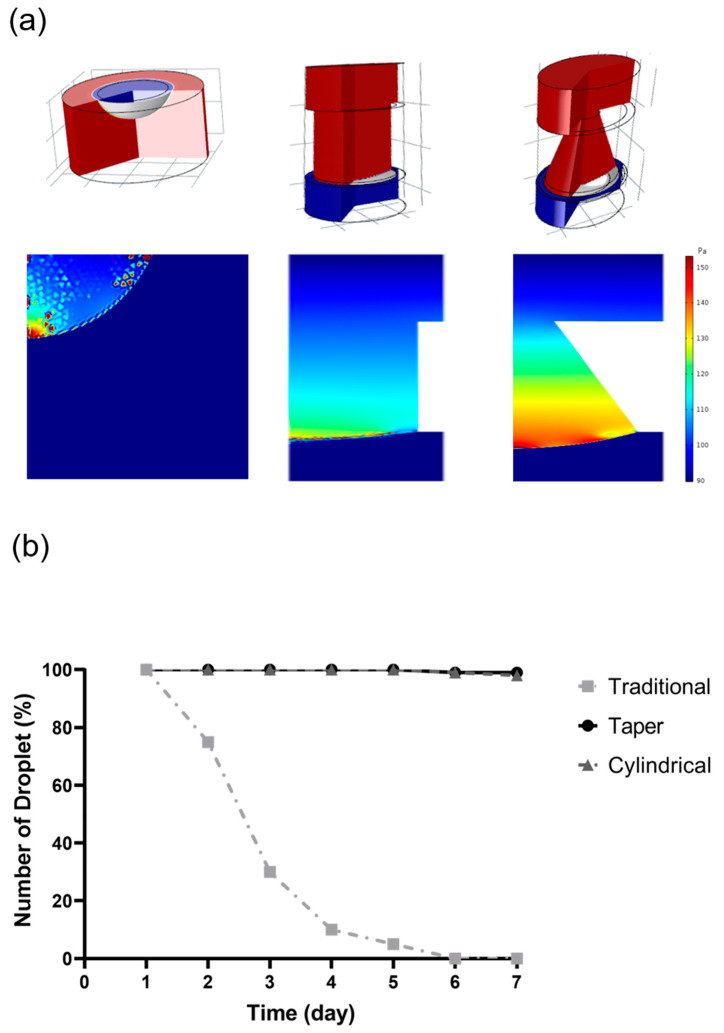
Droplets stability test: traditional hanging drop method, cylindrical tube microfluid chip, and taper-shaped microfluid chip (**a**) computer simulation, and (**b**) long-term stability test.

A computer simulation was used to improve the stability of the hanging drops during cell culture (Figure 4a). We compared three types of culture conditions: traditional hanging drop culture, cylindrical-tube microfluid chip, and taper-shaped microfluid chip. The simulation used a 2D axisymmetric model, with the level set method used to calculate the change in interface pressure of the water droplet-air.

The flow path pressure state in the droplet is similar to atmospheric pressure; hence, the inlet of the chip was uniformly set to the atmospheric pressure inlet flow path in the 2D axisymmetric model. The flow rate was set to 0.5 cc/h for input into the chip, and the same volume of medium was discharged from the outlet. The simulation results showed that because of the surface tension between the wet wall and the droplet, the droplet counteracted the effect of gravity by the surface tension force to maintain a stable two-phase interface.

In addition to the inability to perform culture medium exchange, the traditional culture method can only provide a small cultivation space and carries a pressure up to 89.84 Pa. The cylindrical tube microfluid chip with a diversion hole could effectively resisted gravity via surface tension force and increased the medium bearing capacity. The pressure average reached 97 Pa, indicating high droplet stability in cylindrical tube microfluid chip. The stability factors in the hanging drop network were speed of the flow and droplet radii. In the taper-shaped microfluid chip, the suspension unit had a hole with a narrow upper and lower width and the cross-section formed a wedge shape. Using this device, the liquid droplet could have suspended in the suspension unit stably. In the taper-shaped microfluid chip, when the droplet surface tension force and wet wall contact angle were constant, the modified taper-shaped channel increased the droplet surface curvature and the pressure average of the droplets was increased to 98 Pa, it performed even higher droplet stability than in cylindrical tube microfluid chip.

In this study, we also performed a long-term static test for droplet stability at the bottom of the chip against the acceleration effect of mechanical perturbations. The Figure 4b shows a summary of the counts of fallen droplets in each type of design over time. Droplets in the traditional hanging-drop method began to fall from the second day of the test, and droplets falling continued since then. On the fourth day of the test, the number of original droplets was less than 10% of the initial droplet counts. The results of our study show that the microfluid chip with the taper-tube structures performs with an effect that is not significantly different from that with the cylindrical-tube chip, but performs exceedingly better than the traditional method in preventing the fall of the droplets.

### 2.4. Computer Simulation for Spheroid Formation

**Figure 5:**
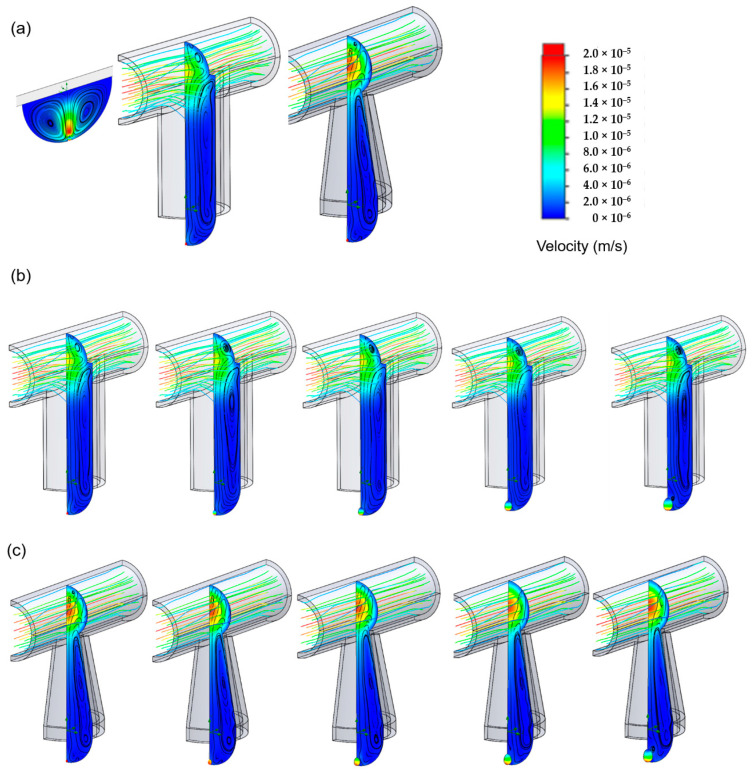
Simulation for spheroid formation. (**a**) Original setting for three culture methods: traditional hanging-drop, cylindrical tube microfluid chip, and taper-shaped microfluid chip; the pressure field exchange on the cell surface during the spheroid formation: (**b**) in the cylindrical tube microfluid chip, (**c**) in the tapered tube microfluid chip.

During simulation, the spheroid diameter was increased from 100 µm to 500 µm using the three different cell culture tray designs (Figure 5). The data were collected post-treatment for the velocity flow from the velocity field flow streamline. The post-process images revealed pressure field coloration on the cell surface, showing that the pressure difference on the cell surface gradually increased as the number of cells increased. The traditional hanging drop method is limited by the lack of medium exchange and insufficient stability of the droplets. The spheroid diameter only grew to 100 µm, and the cell surface force was found to be 11.6 Pa (Figure 5a). The other two microfluid chips that were involved in the process of cell enlargement mimicked the medium exchange process on the cell surface (Figure 5b,c). After extracting the relative pressure and relative force data for the cell surface, the results showed that as the cell diameter increased, the average relative pressure on the cell surface gradually decreased from 11.6 Pa to 9.7 Pa. As the diameter increased, the pressure at the top of the cell gradually decreased, reducing the average pressure. Following the result of the solution exchange experiment, droplets stability test, and computer simulation for spheroid formation, we demonstrated that the taper-shaped microfluid chip was better than the cylindrical tube microfluid chip. We used the taper-shaped microfluid chip to do the follow-up experiment and compare the cell culture effect with the 2D culture method and the traditional hanging-drop method.

### 2.5. Cell Spheroid Formation and Morphology

**Figure 6:**
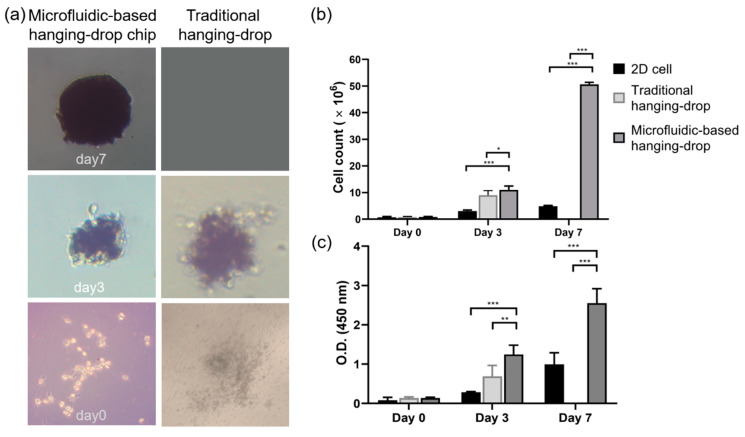
(**a**) Spheroid formation in two kinds of hanging drop methods, (**b**) cell proliferation assay; and (**c**) evaluation of cell activity by BrdU assay; *, *p* < 0.05; **, *p* < 0.01; ***, *p* < 0.001.

In the experiment of spheroid formation, we used the taper-shaped microfluid chip compare with the traditional hanging-drop culture method to evaluate the effect of single WJ-MSCs aggregate to form cell spheroids. As shown in Figure 6a, the first-day WJ-MSCs scattered at the bottom of the drop after seeding both in the chip and in the traditional culture method. Then, cells were aggregated into irregular shape spheroids by day 3. Cell morphology and average diameters of the spheroids were measured by light microscopy. As Figure 6a shown, on day 3 after culturing, the cell spheroid showed a diameter of 200 µm, the diameter of spheroids between the chip or the traditional culture method was no significant difference. However, the images showed that the WJ-MSCs cultured in the chip aggregated more tightly and had smoother surfaces. By day 7, the diameter of spheroid cultured in the chip had reached 500 µm (Figure 6a). However, the traditional culture method is on the limitation of not able to exchange the culture medium, the cell spheroids could not culture to day 7.

### 2.6. Cell Proliferation

In this study, cells were cultured in a 2D microenvironment, the traditional hanging-drop culture method, and 3D microfluidic-based hanging-drop chips. The recovery of the 3D spheroids from the microfluidic culture chips was critical for subsequent analysis. To determine the physical and biochemical changes in the spheroids, spheroids were removed directly from the microfluid chip using a pipette by aspirating a volume of 2–3 mL; a similar method was described by Cavnar et al. [22]. We harvested cells from the 2D culture method and the spheroids from the other two 3D culture methods on days 1, 3, and 7. Cells and spheroids were dissociated into single cells by incubation with 0.25% trypsin-EDTA for 10 min, and then the results of WST-1 assay (Figure 6b) was performed to evaluate the proliferation rate.

The recovery of the 3D spheroids from the microfluidic culture chips was critical for subsequent analysis. To determine the physical and biochemical changes in the spheroids, spheroids were removed directly from the microfluid chip using a pipette by aspirating a volume of 2–3 mL; a similar method was described by Cavnar et al. [22]. We harvested cells from the 2D culture method and the spheroids from the other two 3D culture methods on days 1, 3, and 7. Cells and spheroids were dissociated into single cells by incubation with 0.25% trypsin-EDTA for 10 min, and then the results of WST-1 assay was performed to evaluate the proliferation rate. According to the WST-1 results, on the third day after cell seeding, the WJ-MSCs cultured in the microfluid chip showed a similar growth rate, with a 10-fold higher rate of cell proliferation compared to the number of cells after 2D culture. On day 7, this difference in growth rate was found to increase by 50-fold between the WJ-MSCs cultured in microfluidic-based hanging-drop culture chip and 2D culture environment. The traditional hanging-drop culture method was limited by cannot exchange the culture medium, the cells cannot culture in this method for 7 days.

The results showed that the our microfluidic-based hanging-drop culture chip provided a continuous culture fluid replacement environment and created a good growth environment for the cells. In light of the previous results, we designed some experiments as follows for the microfluidic-based hanging-drop culture system to evaluate the maintenance of the characteristics of WJ-MSCs, including stem cell phenotype, and differentiation to other tissue.

### 2.7. Live/Dead Evaluation

**Figure 7:**
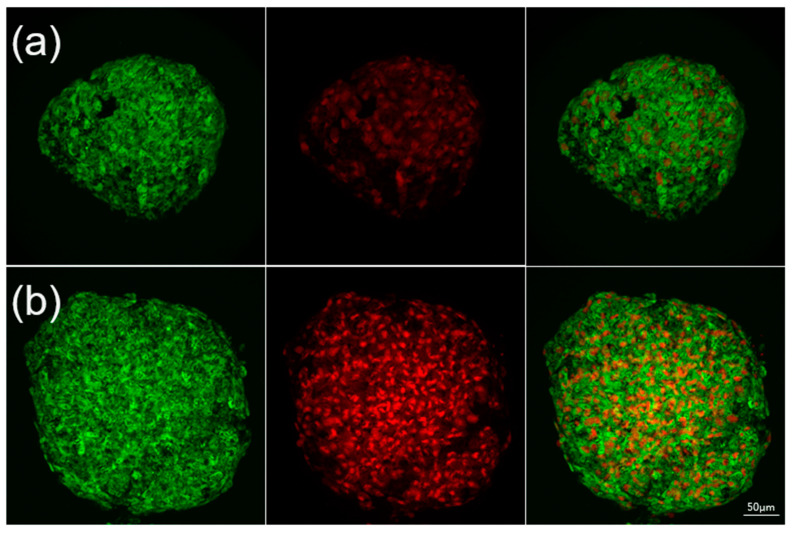
Live and dead stain of the spheroids were cultured in microfluidic-based hanging-drop chip: (**a**) day 3, and (**b**) day 7.

To verify the viability of the cells forming the spheroids, cells were stained with the fluorescent dyes calcein AM and propidium iodide (PI). On day 3, 90% of the WJ-MSC cells cultured in the microfluid chip remained survive (Figure 7a). More importantly, most of the cells cultured in the microfluid chip were stained green on day 7; during this time, cell death rate was below 40% (Figure 7b).

As mentioned in the previous section, WST-1 is a measurement of cell metabolic activities. Thus, we used the BrdU assay to detect the newly synthesized DNA during cell proliferation (Figure 6c). The OD value showed that on the third day after cell seeding, the spheroids cultured in both two 3D culture methods provided with the number of BrdU positive cells exceeded the cell number of the 2D culture method. On the seventh day, the OD value of WJ-MSCs cultured in the chip with a 3-fold higher rate of cell proliferation compared to the number of cells after 2D culture. It showed statistically significant differences in two culture methods (*p* < 0.001). The results showed that the microfluid chip created an environment that allowed growth of stem cell by continuously infusing nutrients and removing waste with a perfusion pump. This culture system demonstrated the ability to maintain cell viability.

### 2.8. Cell Markers

**Figure 8:**
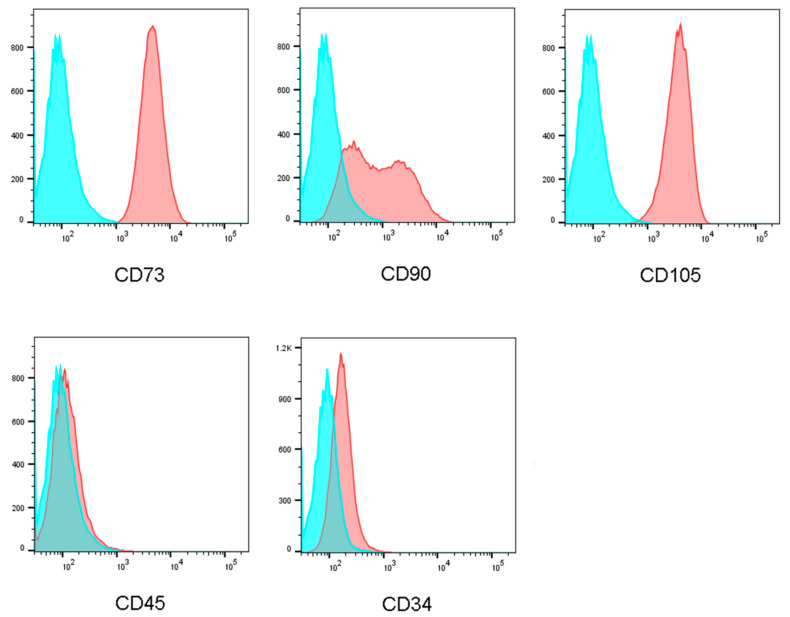
Flow cytometry assay of the WJ-MSCs. The cells expressed CD 73, CD 90, and CD 105, which are surface markers representing mesenchymal stem cells.

The phenotype of the fresh WJ-MSCs was assessed by flow cytometric analysis to detect the expression of CD73, CD90, and CD105 and the non-expression of CD34 and CD45. Following excitation at 488 nm, green fluorescence emission (530 nm/30 nm band pass) and red fluorescence emission (650 nm long pass) were measured. Spheroids harvested on day 7 after cell seeding were treated with trypsin to separate the spheroids into single cells to determine if these cells had maintained their phenotype. The cell markers on the WJ-MSCs showed the same distribution with a pilot study, confirming that the microfluidic culture chip maintained the original stem cell phenotype (Figure 8).

### 2.9. Mineralization Assay

**Figure 9:**
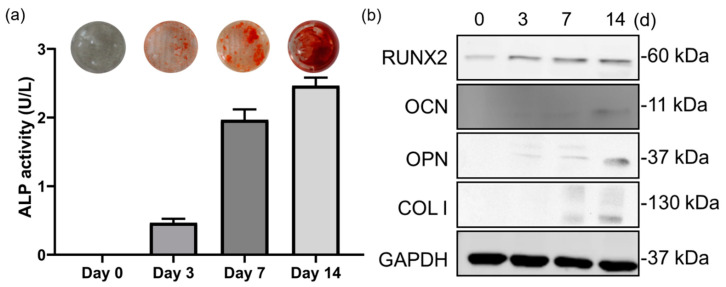
Evaluation of the osteogenic differentiation of the WJ-MSCs. (**a**) Alkaline phosphatase assay and Alizarin red stain, (**b**) western blot for osteogenic markers.

In this study, alkaline phosphatase (ALP) activity was used to indicate the early osteogenic differentiation of WJMSCs. The result was shown to the ALP activity increase after 3 days of osteogenic treatment and reached the highest value on day 14. On day 3, the ALP activity was 0.5 U/L, to the day 7, it increased to 2 U/L, then the maximum value was shown on day 14, increased to 2.4 U/L (Figure 9a). In addition, we also used Alizarin red stain (ARS) to evaluate the capability of cells to differentiate and form the mineralizing matrix. After seven days of osteogenic differentiation treatment, in contrast with the control, the mature osteoblasts differentiated from WJMSCs show intense red-orange staining of mineralized bone matrix (Figure 9a).

### 2.10. Western Blot

The marker expression was evaluated on days 1, 3, 7, and 14 after osteogenic differentiation treatment of WJMSCs. The result showed that the osteogenic markers—including osteopontin (OPN), osteocalcin (OC), collagen I, and the marker runt-relater transcription factor 2 (RUNX2)—were significantly upregulated with osteogenic differentiation treatment compared to the original group on the day 14 (Figure 9b).

## 3. Discussion

Cell therapy refers to a technique in which human cells are used to repair the structure and function of the damaged cells or tissues of a human body as well as treat diseases. To obtain enough cells for cell therapy, studies have been conducted to develop methods for effectively culturing the cells on a large scale. Various methods has been developed to culture cells in a 3D environment, such as by using artificial scaffold [24], non-adherent surface methods [25], spinner flasks, and stirred-tanks [26]. However, artificial scaffolds could cause immunogenic reactions when used in therapy. Additionally, their mechanical properties, mesh size, degradation rate, and chemical properties could alter the cell phenotype, limiting their application [27]. In non-adherent surface methods, ultra-low attachment plates are coated with an inert substrate to prevent cell attachment to the surface of the wells, causing cells to aggregate into spheroids [28]. However, the size and composition of the spheroids cannot be controlled in this method, which is inconvenient for many biomedical applications [29,30]. Other methods as spinner flasks and stirred-tanks require large amounts of materials for culture and space to incubate. Due to shear stress, these methods result in harmful effects on cell physical and biological properties [31]. Other methods for 3D culture involve hanging drop methods, which induce spheroid formation, and microfluidic methods. As cells naturally adhere to each other, the hanging drop method does not require artificial scaffolds; however, it is still limited by the lack of medium exchange during culture [32]. The droplets are also easily detached from the culture plate, resulting in experimental failure. Recently, studies found in microfluidic platforms, living cells can be cultured while nutrients continue to be released [33]. However, it suffers from incapability for large-scale culture, even though it is convenient for drug delivery testing [34].

There have been many microfluid technologies combined with hanging-drop for cell culture developed in the past decade. A common problem within these devices is the cell suspension is centralized introduced at the inlet, which would cause the number of cells in each microwell to fail to regularize. Also, the instability of droplets during the spheroid formation remains an unsolved ordeal. In this study, we successfully established a taper-shaped hanging-drop which was based on the microfluid culture platform for growing uniform-sized cell spheroids. There were four major features in this microfluidic-based hanging-drop culture system. First, the taper-tube design could have increased the stability of droplets, and enhance the rate of liquid exchange. Second, there is a ring surrounding the taper-tube to hold the cells, so we can seed the cell according to the amount we need before perfusion. Third, during the period of cell culture, the mechanical force around the cell was relatively small, which could stably prevent stem cells from differentiating and maintain the phenotype. Finally, cells were designed to accumulate at the bottom of the droplet because of this hanging system, which is convenient for observation or doing other analysis experiments. Details will be described in the following paragraphs.

Compared to other microfluidic-based culture systems [10,35,36], in this study, we used the material, poly(methyl methacrylate) (PMMA), which can enable rapid prototyping and low-cost microfluidic technology. PMMA evaporates into gaseous compounds during CO_2_ laser cutting, so it can cut very cleanly and it is very easy to cut into a complex design for various applications. The thermoplastic feature means PMMA is used extensively in the bio-chip manufacturing process and it provides moderate biocompatibility for different kinds of cells to form spheroids.

A significant advantage of this system was the design of the culture chip with taper-tubes for hanging-drop formation. When the liquid was applied through an inlet, it was drawn through the network by capillary forces along the rims to the culture channel. The channel bottom of the chip contained six hanging culture areas connected by one medium channel though a taper-tube. Droplets that were formed below the taper-tube areas increased with the liquid pressure. The drop size was determined by the surface tension at the liquid–air interface and lateral distance of the rims at the bottom. Frey et al. [10]. observed that based on the surface tension at the liquid–air interface, the hanging drops would spontaneously equilibrate volume. In contrast to other microfluid culture systems, the tray for cell suspension on this microfluid chip shows a taper-shape in the cross-section that provides greater surface tension to resist gravity, which enabled the droplet to be suspended more stably in the chip. By using computer simulation, we first analyzed the droplet stability during droplet formation. The result of simulating in droplet stability showed a similar tendency. The stability factors in the hanging drop network were the speed of flow and droplet radii. The computer simulation results showed that the taper-tube culture method used a deeper flow tube and had a lower curvature interface that caused higher stability of droplet [10,37]. In the taper-shaped microfluid chip, the suspension unit had a hole with a narrow upper and lower width and the cross-section formed a wedge shape. Using this device, the liquid droplet was stably suspended in the suspension unit. In the taper-shaped microfluid chip, when the droplet surface tension force and wet wall contact angle were constant, the modified taper-shaped channel increased the droplet surface curvature, showing high droplet stability. The simulation results also showed that because of the surface tension between the wet wall and the droplet, the droplet counteracted the effect of gravity by the surface tension force to maintain a stable two-phase interface. In this study, we also performed a long-term static test for droplet stability at the bottom of the chip against the acceleration effect of mechanical perturbations. The test results showed that the microfluid chip with taper-tube structures performs exceedingly better than the traditional method in preventing the fall of the droplets.

The cell culture system should be robust enough to withstand against sudden perturbations in a long-term culture period to keep the hanging drops stable. Another advantage of this system is that a ring surrounding the taper-tube could enhance the stability of the droplet. Previous studies showed that micro-ring structures significantly enhance the stability of droplets [38]. Besides, the ring structure could prevent droplets merged with neighboring drops from spreading out [39]. Compared to other designs, the combination of a taper-shaped design and micro-ring structure physically allows for a bigger volume of liquid to be held in the hanging drop and remain stable.

As the result of the 3D modeling method was used to simulate the medium exchange process, the effectiveness and the efficiency of the medium exchange process were determined by observing the volume fraction changes in moisture in the simulation area at different time points. The medium reached the bottom of both types of culture trays by natural convection and free diffusion under the condition of 0.5 cc/h medium exchange. As shown in the curve of the water volume fraction as a function of time, the taper-shaped tray was relatively efficient. For liquid exchange, the effectiveness and the efficiency of the medium exchange process were determined by observing the volume fraction changes in moisture in the simulation area at different time points [37]. Combined with the peristaltic pump, the taper-shaped tray was found to be more efficient discharged liquid from the outlet at a constant rate to achieve liquid exchange that can effectively solve the disadvantages of the traditional hanging drop culture method which cannot replace the culture medium.

The previous study revealed that mechanical forces significantly effect cell activity, such as reduced cell proliferation, mitochondria vacuolization, or the cell cytoskeleton depolymerization. Among these mechanical forces, shear stress and hydrostatic pressure directly affect the physical and biological characteristics of cells and become important participants in cell viability [40,41]. In the final part of the simulation, we mimicked conditions such as hydrostatic pressure, capillary phenomenon, and liquid disturbance during spheroid formation. The condition we set that spheroids diameter were increased from 100 µm to 500 µm in three different cell culture plate designs. As the diameter of the cell spheroid increased, the pressure at the top of the battery gradually decreased, and the average pressure decreased [40]. As a result of surface pressure, a smaller droplet contains a higher pressure than pressure in a larger droplet. Our taper-shaped device could provide a wider surface area for droplets. In such a case, the droplet could be bigger as well as being able to store more solution, and it could benefit the cell culture experiment as it minimizes interference to the cells inside the droplets. Thus, this kind of culture platform is very suitable for shear-sensitive cells—for example, stem cells.

Another mechanical factor which would effect cell activity is the stress on the spheroid surface. According to the result of computer simulation, liquid cohesion in the tapered trays was high enough to form spheroids that remained in the chamber for up to 7 days during culture. Compared to previous studies of the traditional hanging drop culture method, and the other design for microfluid culture method, the taper-shape microfluid chip used to grow cells showed that a higher surface force was associated with an increased spheroid diameter. As the result, the pressure on the spheroid varied with spheroid diameter. For WJ-MSCs, in the early stage of the cell spheroid growth, the surface pressure was high, resulting in high cell cohesion and aggregation. During the middle period to the late period of spheroid growth, the pressure was reduced, enabling rapid cell growth and amplification [42,43,44]. According to a previous study, increased pressure on the cell culture environment may promote the ossification of the MSCs [45]. Therefore, the microfluidic hanging drop chip we developed may to be superior to other culture methods in maintaining the stem cell phenotype.

Besides, in most of the microfluidic-based culture systems [46,47,48], the cell suspension is introduced at the inlet. After the suspension fills the device, the cells take several minutes to deposit on microwell bottoms. The disadvantage of this approach is that the number of cells in each microwell would not be regularize. In this study, as Figure 1 showed, our design was granted with a specialty, before the perfusion pump drives the medium flow, we were able to seed the number of cells we need in each microwell. With a ring at the bottom of taper-tube, our system can load cells and avoid cells from falling or being washed away by the flow to make sure the cell counts that we needed were successfully cultured.

The concept of culture chip is similar to the microfluidic hydrogel hanging-drop network which design by the group of Dr. Frey, the culture system in this study would be ensuring cell viability and original phenotype. More importantly, through scaffold-free culture, cells would tightly be integrated with each other, which enables the simulation to the situation in the human body. During cell spheroid growth, it would be affected by diffusion nutrients, oxygen, and metabolites. To prevent necrotic damage to the spheroid core, high-throughput fabrication of spheroids with a defined size and composition is critical [49,50]. We used the WJ-MSCs as a model to analyze cell spheroid formation in the taper-shaped microfluid chip. A multicellular spheroid shows gradients in nutrients, metabolites, and oxygen along the spheroid radius [51]. The gradient often results in the formation of larger spheroids with a necrotic core [52]. In spheroids above a critical size of 400–600 µm diameter, the innermost cells may die of apoptosis or necrosis. In this chip, spheroids formed on day 3 after cell seeding and grew to approximately 500 µm diameter by day 7. In this study, the WST-1 assay and the BrdU assay were performed to evaluate the proliferation rate. The WST-1 is the measurement of cell metabolic activities. Thus, we used the BrdU assay to detect the newly synthesized DNA during cell proliferation. As the result, most cells cultured in the microfluid chip were found to be alive on day 7, having a cell death rate of less than 40% and showing high proliferation. To verify the ability of this system to maintain the phenotype of WJ-MSCs, before the experiments, fresh WJ-MSCs were assessed for their phenotype to detect the expression of CD73, CD90, and CD105 and the non-expression of CD34, and CD45 [8,53]. After seven days of cell seeding, we analyzed single cells from the spheroids to ensure that their phenotype was maintained. Cell markers on the WJ-MSCs cultured in the microfluidic hanging drop chip showed the same distribution before culture and after culture, demonstrating that the chip maintained the original stem cell phenotype [52,54].

In addition to maintaining the phenotype of the cell spheroids, this culture system also can maintain the character of stem cells that can differentiate into other tissues. In this study, the capabilities of cell spheroid osteogenic differentiation as an indicator to maintain the characteristics of WJMSCs. To determine the osteogenic differentiation ability, the WJ-MSCs were seeded into a microfluidic hanging-drop dish for 3D spheroid culture and start to osteogenic differentiation after three days. In this study, we used alkaline phosphatase (ALP) assay [51], an early marker of osteogenesis, which was found to increase during the differentiation process from the day 7 to the day 14. As the results showed, the ALP value of WJMSCs differentiation on day 14 was 4-fold higher than the value on the day 3. Compared with other literature, we can see the same tendency of ALP value [55]. In addition, we also used Alizarin red stain (ARS) to evaluate the capability of cells to differentiate and form the mineralizing matrix. After seven days of osteogenic differentiation treatment, in contrast with the control, the mature osteoblasts differentiated from WJMSCs showed intense red-orange staining of mineralized bone matrix. Alizarin red staining for the late stages of osteogenic differentiation showed the capability of the spheroids which formed by the microfluidic hanging drop chip, to differentiate and form the mineralizing matrix. MSCs differentiation to osteoblasts requires the expression of an essential transcription factor, Runt-related transcription factor 2 (Runx2) [56,57], which is considered to be the master gene for osteoblast differentiation, can lead to osteoblasts entering the mineralization cycle required for bone formation [58]. Osteocalcin (OCN) [59], osteopontin (OPN) [60], and collagen I (Col-I) [58] were seen as the bone markers during osteoblast differentiation [61]. The western blot evaluated the marker expression on days 1, 3, 7, and 14 after osteogenic differentiation treatment. Firstly, the effect of Runx2 expression on osteogenic differentiation was examined. As expected, the data of Runx2 expression showed since day 3. Inductive levels of OCN and OPN were increased from day 3 till day 14. An increase of collagen I was also shown at 7 and 14 days.

The above experiments proved that, in addition to maintaining the stem cell phenotype, this micro-fluidic hanging drop culture system also could had retained the characteristics of stem cells that can be differentiated into other tissues. Furthermore, according to previous literature, different sizes of spheroids have been used as in vitro models for drug testing or implanted into an animal to recapitulate the growth and proliferation of tumor tissues [62,63]. The chip we developed in this study exhibited flexibility for hanging-drop networks, enabling spheroid formation under different conditions on the same chip. When the perfusion pump drives liquid flow, turbulence occurs in the drops; cells in the drop settle to the bottom and begin to aggregate. Under different cultural conditions, this chip can be used for various applications such as drug delivery or as an in vitro platform for cell therapy [4,21,64,65]. In addition to the formation and culture of the spheroids, how to observes and analyze the spheroids has been gradually being valued. Researcher Torisawa et al. designed a microfluid chip containing pyramid-shaped microwells, let spheroids can be scan by electrochemical microscopy on the chip [66]. Other researchers used different materials such as electro-spun PDMS nanofibers [67], or hydrogel [68] to enhance the structure of spheroid and assist positioning during the test. The design of the device in this study was made according to the specifications of a standard cell culture plate which was widely used in biological experiments. It cooperated with general detection platforms on the market, such as ELISA readers. We developed this perfusion cell culture device with a rapid prototyping method to create it in a simple way, and we saved a lot of production costs and still remained modifiable according to experiment needs. The device can be installed with other devices in the future, such as photoacoustic detection equipment or magnetic field stimulation system.

## 4. Materials and Methods

### 4.1. Microfluid Chip Design and Fabrication

The microfluidic hanging-drop chip has been shown in Figure 1a. The microfluid structure is divided into three layers: the upper layer contains four sets of 2 mm wide medium channels as inlets and outlets, using the laser (XS-300, Eurolaser GmbH, Lüneburg, Germany) to ablate the holes; the middle layer, using the laser to ablate the channel of liquid flow and the lower layer, contains six hanging culture areas, connected to one medium channel though a taper-tube. Taper-shaped holes were made via three-axis CNC (EGX-400, Roland Inc., Hamamatsu, Japan) grinding to make the slope. The design has been shown in Figure 1b. The surface roughness of PMMA-based microfluid chip was 210 nm through laser ablation, and R_a_ = 1100 nm was engraved via CNC. The angle of the taper-shaped structure is 10–15 degrees.

All consumables in the microfluid chip were sterilized by treatment with ethylene oxide. The chip was installed inside an incubator with humidified air (37 °C, 5% CO_2_) with a peristaltic pump which provided continuous medium replenishment.

### 4.2. Computer Simulation for Droplet Stability and Medium Exchange

The software COMSOL Multiphysics^®^ (COMSOL, Inc., Stockholm, Sweden) was used as a simulation tool in this study. We compared three different culture methods: the traditional hanging-drop method, the cylindrical-tube culture method, and the taper-tube culture method. To ensure the stability of the water droplet, we used a 2D axisymmetric calculation model and a level set method with similar culture medium density, medium flow rate, and balance between gravity, buoyancy, and drag forces, and then tracked the position of the interface between the two immiscible fluids. The effects of surface tension and gravity were also evaluated. In the experimental state, the culture solution was allowed to flow through the tube at a velocity of 0.5 cc/h. To equilibrate the computing time and simulation precision, a zero static pressure boundary condition was set in the inlet.

To understand the efficiency of medium exchange during cell culture, 3D modeling was performed with a finite volume method to simulate two-phase flow. This simulation is performed in 3D symmetry model, which assumed the water–air interface is a slip wall boundary. A symmetry plane is used to reduce the computation time, an inflow boundary and an outflow boundary with 1 × 10^−6^ l/min volume rate on both side of the tube. Chips with cylindrical tube and tapered tube were compared at a simulated flow rate of 0.5 cc/h. A single culture tray was utilized in the medium exchange process.

### 4.3. Droplet Stability and Medium Exchange in the Chip

We also observed the movement of methyl blue (Sigma-Aldrich, St. Louis, MO, USA) within the chip to verify medium exchange. The microfluid chip was initially filled with water, and then water containing methyl blue was injected into the chip from the inlet at a rate of 0.5 cc/h. Water stained with methyl blue filled the tube form inlet by capillary action, and then replaced the original water in the tray because of the difference in densities between the two liquids and through free diffusion. At the same time, the peristaltic pump discharged the liquid from the outlet at a constant rate to achieve liquid exchange.

A long-term static test was performed for droplet stability at the bottom of the chip against the acceleration effect of mechanical perturbations [39]. Droplets of 50 µL cell culture media were formed in 10 hanging drop sites located at the traditional hanging-drop method plate and in the two designs of the microfluidic-based hanging drop culture chips. Three plates were each subsequently placed on a waver shaker with 10 s of ramp time and 30 degrees of the slope to ensure each droplet experiences different forces of centripetal acceleration to find the best design for the droplet stability.

### 4.4. Computer Simulation for Spheroid Formation

In this study, we also simulated the cell spheroid formation in the microfluid chip. A flow rate of 0.5 cc/h was applied from the inlet, and the same volume of medium was discharged at the outlet. Hanging-drop spheroids with sizes of 100–500 µm diameter were evaluated to examine the pressure and surface forces during a medium exchange and their effects on the cell spheroid formation.

### 4.5. Cell Culture and Self-Assembly of Spheroids

To determine the spheroid formation and culture capabilities of the microfluid chip, the WJ-MSCs (Bioresource Collection and Research Center (BCRC, Hsinchu, Taiwan)) from passages 3–6 were used in all experiments.

In this study, two culture methods were used to compare the experimental results between 2D and 3D culture. The cells were seeded into a 10 cm dish at a density of 10^6^ cells/mL for 2D monolayer culture and allowed to adhere. Cells were cultured in a Dulbecco’s modified Eagle medium (Gibco BRL, Grand Island, NY, USA) containing 2.2 g NaHCO_3_ (Sigma-Aldrich, St. Louis, MO, USA), 10% fetal bovine serum (Sigma-Aldrich, St. Louis, MO, USA), and 1% penicillin/streptomycin (Sigma-Aldrich, St. Louis, MO, USA) in each 1 L culture medium and under 5% CO_2_ at 37 °C. The culture medium was replaced with fresh medium every 48 h.

For 3D spheroid culture, the microfluidic hanging drop chip was used. Before the experiment, the chip was sterilized under UV light for 30 min then filled with culture medium. Cells were added at the same density as in 2D culture and cultured under the same conditions. Cell loading was performed using conventional 100 mL pipettes into the taper-tube of the chip. The medium for 3D culture was exchanged at 24 h after cell seeding using a peristaltic pump at a flow rate of 0.5 cc/h through the inlet and outlet.

### 4.6. Self-Assembly of Spheroid and Cell Proliferative Quantification

After 1, 3, 7, and 10 days of cell culture using the 2D method and spheroid culture on the hanging-drop chip, the cells were harvested to evaluate their proliferative abilities. Cell morphology and spheroid average diameters were measured by light microscopy (CiS, Nikon, Tokyo, Japan).

Cell proliferation was quantified by the WST-1 assay (Merck KGaA, Darmstadt, Germany). The cells were seeded into a culture plate then grown overnight. After washing the cells twice with phosphate-buffered saline, 180 µL culture medium was added, followed by 20 µL of the WST-1 reagent. The cells were incubated at 37 °C for 2–4 h and then absorbance was measured on an ELISA plate reader at a wavelength at 450 nm. Background absorbance collected at 630 nm was subtracted from the final value.

The BrdU Cell Proliferation ELISA Kit (Abcam, Cambridge, England) is an analog of the nucleoside thymidine used in the BrdU assay to identify proliferating cells. The cells were harvested on the third day and on the seventh day after cell seeding, from 2D culture and microfluidic hanging- drop culture. The cells were seeded into a black 96 well plate, cell count was 2 × 10^4^ cells per well, then incubated overnight. The BrdU solution was added to the plate wells, and cells were incubated for 2 h and then the medium was removed. For suspension cells, plate was centrifuged at 300× *g* for 10 min, then the medium was removed. 100 µL of the fixing solution was added per well and the plate was kept at room temperature for 30 min. 100 µL of the primary antibody solution was added into each well and the plate were kept at room temperature for 1 h. Plates were washed three times and 100 µL of the HRP-conjugated secondary antibody solution was added into each well and the plates were kept for 30 min and then 100 µL TMB substrate was added. Finally, 100 µL of the stop solution was added. ELISA plate reader was used at a wavelength of 450 nm. Background absorbance collected at 600 nm was subtracted from the final value.

### 4.7. Live/Dead Evaluation

After culturing for 1, 3, 7, and 10 days, the viability of the spheroid cells was assessed using a LIVE/DEAD kit (Sigma-Aldrich, St. Louis, MO, USA). The cells were stained with 4 µM Calcein AM and 4 µM PI for 30 min in 37 °C. Calcein-AM broken down by esterase in a viable cell results in a strong green fluorescence signal (excitation: 490 nm, emission: 515 nm), whereas dead cells are stained as red by the aqua-fluorescent reactive dye PI (excitation: 535 nm, emission: 617 nm). Cell survival was observed with a laser confocal microscope (LSM 700, Zeiss, Oberkochen, Germany), and 3D cell images were reconstructed.

Cells and spheroids were dissociated into single cells by treatment with 0.25% trypsin-EDTA for 10 min. The cell survival rate was also quantified by the CCK-8 assay (CCK-8, Dojindo, Kumamoto, Japan). The cell suspension (100 µL; 5000 cells/well) to a 96-well plate and the plate was incubated for 24 h in a humidified incubator. After adding 10 µL of the CCK-8 solution to each well and incubation for 2 h, cell proliferation was observed with a TECAN 200/200Pro multimode microplate reader (TECAN Trading AG, Männedorf, Switzerland).

### 4.8. Cell Markers

Previous studies have shown that the phenotype of stem cells is altered when the cells are cultured in large numbers. Before using the WJ-MSCs in the experiments, the fresh cells were assessed by flow cytometric analysis to detect the expression of CD73, CD90, and CD105 and the non-expression of CD34 and CD45. A single-cell suspension (1 × 10^6^ cells/mL, 1 mL) was stained with antibody for 30 min at 4 °C in the dark and centrifuged for 5 min at 1600 rpm. The stained cells were analyzed by flow cytometry (LSRFortessa X-20, BD Biosciences, Franklin Lakes, NJ, USA) at an excitation of 488 nm. The green fluorescence lines represent the secondary anti-mouse IgG control, emission (530 nm/30 nm band pass), and the corresponding CD marker expression, red fluorescence, emission (650 nm long pass) were measured.

To determine the ability of the culture system to maintain the original phenotype of the WJ-MSCs, we harvested the spheroids after culturing for 7 days and used trypsin to separate the spheroids into single cells for flow cytometry analysis.

### 4.9. WJMSCs Treat for Osteogenic Differentiation

To determine the capabilities of spheroid osteogenic differentiation, the WJ-MSCs from passages 3–6 were seeded into a microfluidic hanging drop chip at a density of 10^6^ cells/mL for 3D spheroid culture as stated above. For osteogenic differentiation, after 3 days of culturing the spheroids, the culture medium was replaced with osteogenic differentiation medium (Thermo Fisher Scientific Inc, MA, USA) and kept for 14 days. Cells cultured in differentiation medium were evaluated for proliferation on the 3rd day; alkaline phosphatase (ALP), an early marker of osteogenesis, on day 7; osteogenic gene expression on day 14 and alizarin red S staining for the late stages of osteogenic differentiation.

### 4.10. Alkaline Phosphatase Activity Determination

After osteogenesis treatment of WJMSCs, the spheroids were washed with PBS and lysed into single cell with 0.25% trypsin-EDTA. Intracellular ALP assays (Abcam plc., Cambridge, UK) were performed on days 0, 3, and 7 post-treatment. 1 × 10^5^ cells were harvested, washed with cold PBS, then resuspended in assay buffer. Centrifuge samples at 4 °C at top speed for 15 min and collect the supernatant. 50 µL lysed sample was mixed with 50 µL 5 mM pNPP. Incubate plate at 25 °C for 60 min protected from light then adding 20 µL stop solution to stop the reaction. The absorbance of p-nitrophenol released in the samples was measured at 405 nm, using a TECAN 200/200Pro multimode microplate reader (TECAN Trading AG, Männedorf, Switzerland).

### 4.11. Alizarin Red S Stain for Identify Calcium in WJMSCs Osteogenesis

At day 14 post-treatment, spheroids were lysed into single cells with 0.25% trypsin-EDTA then washed with Dulbecco’s PBS, w/o Ca^++^. Add 70% ethanol for 10 min to fix cells and stained with 40mM Alizarin Red S (Sigma-Aldrich, St. Louis, MO, USA), incubate at room temperature in the dark for 10 min. The cells were carefully washed four times with 1 mL distilled water and PBS was added. The result of the staining was observed by light microscopy (CiS, Nikon, Tokyo, Japan), undifferentiated WJMSCs are slightly reddish, whereas WJMSC-derived osteoblasts with extracellular calcium deposits would be bright orange–red.

### 4.12. Western Blotting Assays

Cells were harvested by scraping in ice-cold RIPA lysis buffer (Sigma-Aldrich, St. Louis, MO, USA). Western blotting was performed and blots were probed with osteogenic differentiation markers, osteopontin (OPN, Abcam plc., Cambridge, UK), osteocalcin (OC, Abcam plc., Cambridge, UK), collagen I (Abcam plc., Cambridge, UK), and the marker runt-relater transcription factor 2 (RUNX2, Abcam plc., Cambridge, UK). Protein bands were detected using a western lightning plus-ECL, enhanced chemiluminescence substrate (PerkinElmer Inc, Waltham, MA, USA).

### 4.13. Statistical Analysis

The Student’s *t*-test was used to compare the means of two independent sample groups. A *p*-value of less than 0.05 was considered to indicate statistical significance. All experiments were conducted at least in triplicate for statistical analysis, and the mean ± standard deviation (SD) has been presented.

### 4.14. Limitation

There were several limitations to this study. (1) The spheroid size was limited by cellular vulnerability to hypoxia in 3D culture; (2) not all of the conditions be examined to various properties that affect the spheroid formation and the spheroid viability; (3) lack of long-term in vivo follow-up data to evaluate spheroid interactions with host cells.

## 5. Conclusions

Compared to the traditional culture methods, our microfluid chip has the following advantages: increased surface area, providing enough space for the cells to attach and grow; the culture environment can be homogenized by regulating gas pressure, pH, and the liquid flow rate; increased connections between cells, reduced shear force on the cell surface during growth; a closed culture environment to reduce the risk of contamination; and an automatic media exchange. Cell spheroids are a concentric arrangement of the heterogeneous cell populations with different cellular activities. Recently, some studies have indicated that modulating the spheroids can enhance their own regenerative capacity. Thus, this microfluid chip can be used as an in vitro platform representing in vivo physiological conditions, and in regenerative therapy.

## Abbreviations

2D	Two-dimensional
3D	Three-dimensional
NIH	National Institute of Health
PMMA	Poly-methylmethacrylate
SD	Standard deviation
WJ-MSC	Wharton’s jelly mesenchymal stromal cell
